# Inhibitory control in addictive behaviors: is there room for memory suppression?

**DOI:** 10.3389/fnhum.2025.1545176

**Published:** 2025-06-05

**Authors:** Eduardo López-Caneda, Natália Almeida-Antunes

**Affiliations:** ^1^Psychological Neuroscience Laboratory (PNL), Research Center in Psychology (CIPsi), School of Psychology, University of Minho, Braga, Portugal; ^2^RISE-Health, Center for Translational Health and Medical Biotechnology Research (TBIO), ESS, Polytechnic of Porto, Porto, Portugal

**Keywords:** inhibitory control, memory suppression, addictive behaviors, substance use disorders (SUDs), cognitive training

## Forgetting as an adaptive human mechanism

“*If we remembered everything, we should on most occasions be as ill off as if we remembered nothing*” (James, 1890). This phrase by James, like many others found in his masterpiece, *The Principles of Psychology*, perfectly encapsulates the idea that forgetting, contrary to common perception, is (in most cases) not a negative phenomenon, but serves essential adaptive functions in human life.

Indeed, forgetting may be essential for mental health, as it helps regulate negative emotions by limiting access to unpleasant or embarrassing memories, thereby fostering subjective wellbeing and emotional resilience (Nørby, 2018). It also plays a crucial role in learning, facilitating the transition from detailed episodic memories to more generalized and efficient knowledge. An extreme example of the challenges associated with remembering vast amounts of information is the famous case of Solomon Shereshevsky, a Russian journalist with an extraordinary memory who was unable to forget irrelevant details, often becoming overwhelmed by excessive mental associations, as beautifully documented by Alexander Luria in the second half of the 20th century (Luria, 1968; also see Fawcett and Hulbert, 2020; Price and Davis, 2008). Moreover, forgetting ensures that our cognitive processing remains relevant to the present and future, as it filters out outdated information, enabling us to adapt flexibly to new situations and make better-guided decisions (Kuhl et al., 2007; Richards and Frankland, 2017). Thus, forgetting proves to be not only a necessary process for maintaining a healthy emotional state but also an essential mechanism for efficient cognition and dynamic adaptation to an ever-changing environment.

## Cognitive and neural mechanisms underlying memory suppression

The examination of the ability to voluntarily suppress memories has garnered increasing interest over the past two decades, partly due to the groundbreaking work by Anderson and Green (2001). In their seminal study, they developed the Think/No-Think (TNT) task, which was adapted from the classical Go/No-Go paradigm to investigate the suppression of unwanted memories. This task, designed to replicate situations in which individuals encounter reminders of unpleasant memories, requires participants to either recall (Think) or suppress (No-Think) paired word items. The study showed that suppressing memories through executive control processes significantly impairs the recall of suppressed items compared to baseline and actively recalled items (Anderson and Green, 2001). Subsequent research has expanded on this work, demonstrating that suppression-induced forgetting extends beyond neutral word pairs to emotional (Noreen and MacLeod, 2013, 2014) and immoral (Satish et al., 2022, 2024) autobiographical memories, motor actions (Schmidt et al., 2023) and even fearful imaginings about the future (Benoit et al., 2016). Additional studies have also identified the neural mechanisms underlying memory suppression, highlighting the role of prefrontal regions such as the dorsolateral prefrontal (DLPC) cortex and the inferior frontal gyrus (Anderson et al., 2004; Apšvalka et al., 2022; Depue et al., 2007; Paz-Alonso et al., 2013), which exert control over hippocampal and parahippocampal activity, inhibiting/preventing the retrieval of memories or the reinstatement of sensory information related to the learned material (Gagnepain et al., 2014; Mary et al., 2020; Schmitz et al., 2017; Yang et al., 2021). This top-down inhibitory control signal from prefrontal regions not only targets the hippocampus but also modulates other brain regions based on the content of the avoided memories, such as the amygdala for emotional content (Depue et al., 2007; Gagnepain et al., 2017) and the fusiform cortex for visual information (Gagnepain et al., 2014). Altogether, this emerging body of research has supported the view of memory suppression as an integral cognitive process within executive functions, specifically within the domain of inhibitory control (Diamond, 2013), sharing common neuroanatomical structures and neural pathways (Castiglione et al., 2019; Depue, 2012; Wessel and Anderson, 2024).

## Memory suppression and addiction: theoretical perspectives and model integration

Unwanted or intrusive thoughts are considered a hallmark of several psychiatric disorders, such as depression and anxiety, posttraumatic stress disorder, and obsessive-compulsive disorder (Clark, 2018; Ehlers et al., 2004; Harrington and Blankenship, 2002; Julien et al., 2007). While limited in number, studies on these clinical conditions suggest a reduced capacity to effectively suppress unwanted thoughts or memories in individuals affected by some of these psychiatric conditions (Catarino et al., 2015; Depue et al., 2010; Diwadkar et al., 2017; Marzi et al., 2014; Storm and White, 2010; Sullivan et al., 2019). However, the role of memory suppression mechanisms in addiction—a clinical condition characterized by maladaptive and persistent substance-related thoughts that often drive compulsive use (Kavanagh et al., 2005)—remains largely underexplored. This gap in research is particularly concerning given the potential implications for understanding and treating addiction, where the inability to suppress maladaptive memories may contribute to the cycle of craving, relapse, and compulsive consumption (Almeida-Antunes et al., 2024b).

**Figure 1:**
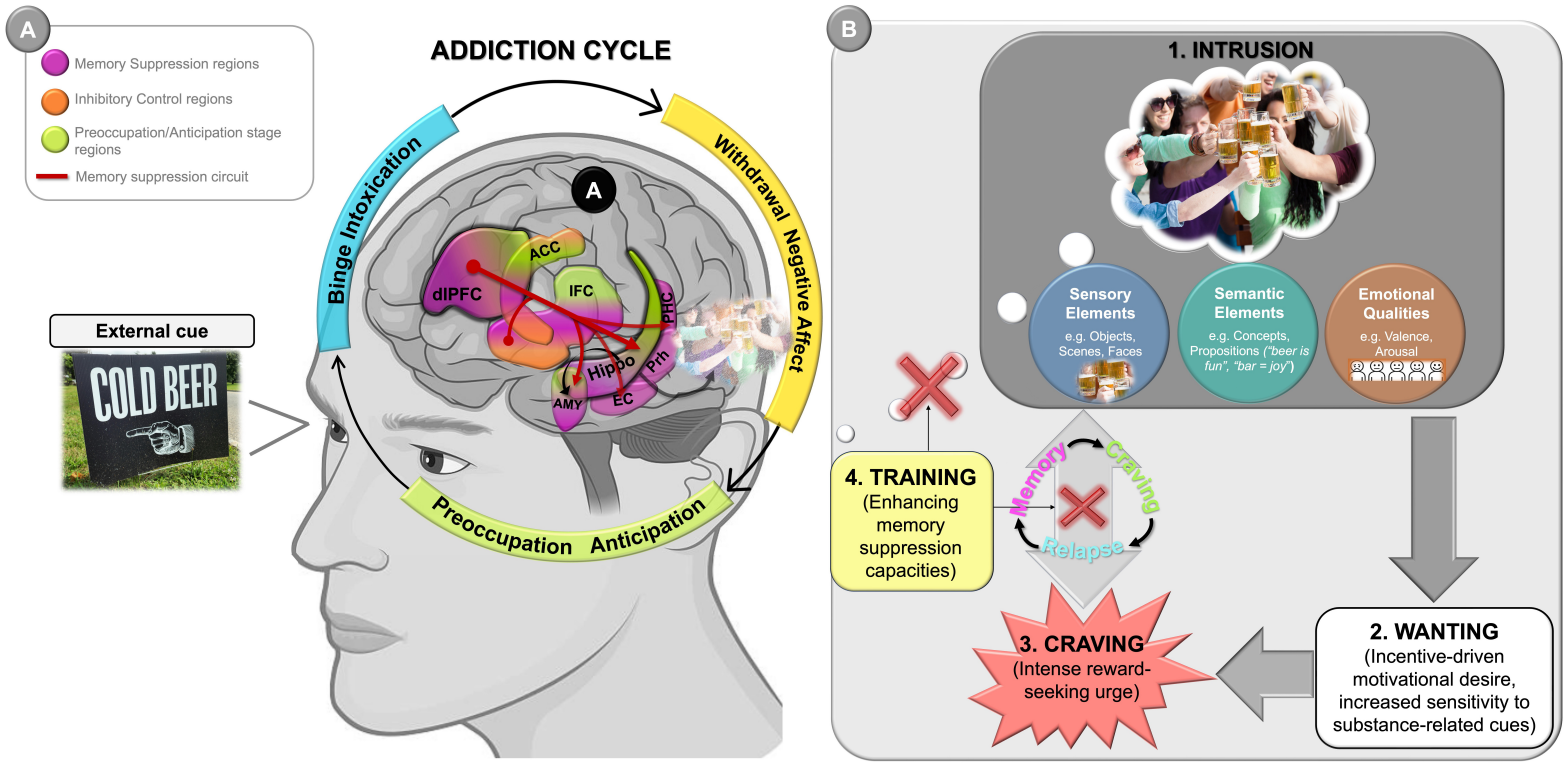
Graphical representation illustrating the role of drug-related memories and memory suppression in the addiction cycle. **(A)** According to the neurobiological model of addiction (Koob and Volkow, 2010), the transition from initial voluntary drug use to compulsive drug-seeking behavior unfolds through a spiraling cycle of three stages—binge/intoxication, withdrawal/negative affect, and preoccupation/anticipation. In the *preoccupation/anticipation stage*, drug-related memories become increasingly salient, contributing to intrusive thoughts, strong craving, and heightened reactivity to substance-related cues. These processes are compounded by impaired executive control—particularly dysfunctions in prefrontal regions—undermining not only the ability to inhibit drug-seeking behavior but also the capacity to suppress recurrent, intrusive substance-related thoughts via the memory suppression circuit. **(B)** The conceptual framework of the *preoccupation/anticipation* stage closely aligns with the Elaborated Intrusion (EI) Theory of Desire (Kavanagh et al., 2005; May et al., 2015). According to this theory, craving is triggered when an initial intrusive thought—often a brief, automatic cognitive or sensory representation of the substance—emerges into consciousness. These intrusions are typically reactivated by internal (e.g., affective states, bodily sensations) or external cues (e.g., environments, people, or images associated with previous drug use), as illustrated in the photograph on the left, in part A, and are then progressively elaborated into vivid, emotionally charged mental images, often reflecting episodic memories—such as toasting with a beer at a party with friends [as depicted in **(B.1)**]. Once elaborated, such memory episodes may evoke *wanting*—that is, an incentive-driven motivational desire (Robinson and Berridge, 1993; Berridge and Robinson, 2016)—which manifests as heightened reactivity to substance-related cues and increased salience of drug-associated goals **(B.2)**. This may, in turn, escalate into *craving* **(B.3)**, experienced as an intense, reward-seeking urge that captures attention, biases decision-making, and promotes substance-seeking behavior—ultimately reinforcing the memory-craving relapse cycle. Enhancement of memory suppression capacities could eventually reduce the accessibility and impact of drug-related intrusions, thereby weakening craving episodes and lowering the risk of relapse **(B.4)**. Photographs were obtained from the *Alcohol, Tobacco, and Other Drug Public Domain Photo Database* of the *Journal of Studies on Alcohol and Drugs* (left image), and from the *Galician Beverage Picture Set* (López-Caneda and Carbia, 2018; right image). ACC, anterior cingulate cortex; AMY, amygdala; EC, entorhinal cortex; dlPFC, dorsolateral prefrontal cortex; Hippo, hippocampus; PHC, parahippocampal cortex; PFC, prefrontal cortex; Prh, perirhinal cortex.

This recurring cycle underscores the chronic and progressive nature of substance abuse, which is commonly understood as a condition that evolves from impulsive to compulsive behavior. According to one of the most influential neurobiological models of addiction (Le Moal and Koob, 2007; Koob and Volkow, 2010) this transition unfolds through a spiraling cycle of three stages—binge/intoxication, withdrawal/negative affect, and preoccupation/anticipation (see Figure 1A). The *binge/intoxication stage* involves the acute effects of substance use, characterized by the activation of the brain's reward systems leading to the experience of euphoria and the formation of habitual patterns of use. This is followed by the *withdrawal/negative affect stage*, during which the absence of the substance triggers a negative emotional state, including anxiety, dysphoria, and irritability. The persistence of drug-related memories appears to be intimately linked to the *preoccupation/anticipation stage*, which is marked by intensified craving, heightened sensitivity to substance-related cues, and impaired executive control—factors that significantly contribute to relapse (Koob and Le Moal, 2005; Koob and Volkow, 2016). Indeed, evidence suggests that drug-related memories play a crucial role in sustaining drug use and driving high relapse rates in substance use disorders (SUDs), as they can be triggered by drug-associated cues, eliciting cravings, impulsive behaviors and reduced self-control (Milton and Everitt, 2012; Wise and Koob, 2013; Everitt and Robbins, 2016; Hogarth, 2020; Lüscher et al., 2020). Accordingly, the craving phenomenon and drug-related memories are deeply intertwined, reinforcing each other in a self-perpetuating cycle (Ekhtiari et al., 2016). In this sense, craving is a learned response that connects drug use and its context to pleasurable or relief experiences, driving drug-seeking behavior, and can be elicited by external or internal cues, including memory retrieval (see Figure 1B). Consequently, retrieving substance-related memories can trigger the feeling of craving, which may, in turn, evoke further memories linked to consumption (Berridge and Robinson, 2016; Goldstein and Volkow, 2002). This excitatory cycle is grounded in neural mechanisms, with studies showing that both cue-elicited craving and intoxication increase activity in temporal regions, such as the inferior and middle frontal gyrus, as well as the hippocampus—a key region for retrieving drug-related memories—which may further reinforce substance-seeking behavior by facilitating the recall of substance-related memories (Langleben et al., 2008; Li et al., 2012, 2015; Volkow et al., 2004; Wei et al., 2020; Ekhtiari et al., 2016).

In light of this, it can be suggested that the inability to suppress such memories might potentially influence the behavior of individuals with SUDs. These difficulties could represent a key factor underlying the mechanisms involved in the preoccupation/anticipation stage, thereby contributing to the persistence of the addiction cycle (Figure 1A). A closer look at this stage reveals the engagement of a broad neurocircuitry, including regions associated with memory suppression, such as the DLPFC, hippocampus, and amygdala (Koob and Volkow, 2010). Moreover, the reduced prefrontal control inherent to this stage supports the notion of increased retrieval of drug-related memories, as diminished executive function may facilitate the automatic reactivation of these memories, reinforcing cravings and leading to further substance-seeking behavior (Noël, 2024). Specifically, hypofunction of the prefrontal cortex (PFC) may impair its control over memory-related regions, such as the hippocampus and amygdala (Depue et al., 2007; Gagnepain et al., 2017; Yang et al., 2021). Consequently, this exacerbates the occurrence of intrusive substance-related thoughts, which in turn trigger craving, as well as drug-seeking and drug-taking behaviors (Figure 1B). These behaviors perpetuate the memory-craving relapse cycle and drive progression to the binge/intoxication phase.

This conceptual framework aligns with and extends two influential motivational models of addiction: the Elaborated Intrusion (EI) Theory of Desire (Kavanagh et al., 2005; May et al., 2015) and the Incentive Salience (IS) Theory (Robinson and Berridge, 1993; Berridge and Robinson, 2016). According to the EI Theory, craving arises when an intrusive cognitive or sensory representation of the substance is elaborated into a vivid and affectively charged episode. Our proposal suggests that enhancing memory suppression may prevent such intrusions from occurring in the first place, thereby reducing the need for elaboration, and disrupting the craving episode before it consolidates. These intrusions often consist of episodic representations linked to prior drug use in emotionally salient contexts—for example, recalling the feeling of euphoria when taking cocaine in a nightclub, the sound of a beer bottle opening during a barbecue with friends, the smell of cannabis in a specific room, or the image of a particular street corner where one used to buy drugs. Such memories are typically reactivated by sensory or contextual cues and can trigger strong craving responses (May et al., 2015). Importantly, the components of intrusive desire described in the EI Theory—such as affect-laden imagery, sensory impressions, and propositional knowledge about the substance—often emerge jointly through the reactivation of episodic memories. Recent evidence indicates that suppressing such memories may reduce not only their explicit recall but also the accessibility of associated conceptual content (Taubenfeld et al., 2019; Wang et al., 2019). This suggests that memory suppression may not only interfere with the initial intrusion but also limit the availability of semantic knowledge that fuels the elaboration process. For example, suppressing the memory of drinking beer with a close friend in a particular bar may not only reduce access to that specific episodic trace, but also weaken the associated propositional beliefs such as “*beer is fun*” or “*bar* = *joy*,” which could otherwise contribute to the motivational amplification of craving.

In parallel, the IS Theory distinguishes between *liking* (the hedonic value of the substance) and *wanting* (the automatic motivational pull). Thus, it is possible that memory suppression acts specifically on wanting, by reducing the salience and motivational impact of substance-related cues and memories. Accordingly, several studies have showed that suppressing unwanted memories not only impairs later recall of the suppressed material, but also reduces its affective value, attentional capture, and perceptual vividness (Gagnepain et al., 2014, 2017; Harrington et al., 2021; Hertel et al., 2018; Legrand et al., 2020). In this way, the ability to suppress episodic drug-related content—along with the beliefs and semantic associations it evokes—may contribute to modulating the incentive salience of drug-associated stimuli, thereby acting as a cognitive mechanism to attenuate maladaptive motivational responses in addiction.

## Emerging evidence for impaired memory suppression in alcohol misuse

Although evidence has consistently showed that individuals with drug addiction exhibit structural and functional alterations in brain regions involved in executive control—and, by extension, also implicated in memory suppression—(Goldstein and Volkow, 2011; Zilverstand et al., 2018), research on the ability to inhibit unwanted memories in SUDs remains scarce. To the best of our knowledge, only three studies have specifically examined this ability in relation to alcohol consumption patterns. Notably, all three reported impairments in both the neural correlates and/or the behavioral performance underlying the suppression of unwanted memories, including those related to alcohol (Almeida-Antunes et al., 2024a; Nemeth et al., 2014; Simeonov et al., 2022). Specifically, Nemeth et al. (2014) observed that individuals with alcohol dependence exhibited an impaired ability to suppress retrieval compared to healthy controls. Building on these findings, Simeonov et al. (2022) found that hazardous drinkers also had difficulties in suppressing retrieval, but only for alcohol-related associate pairs, suggesting a selective impairment in suppressing alcohol-related memories in this population. Extending this line of research, Almeida-Antunes et al. (2024a) found that young binge drinkers also exhibited difficulties in memory suppression mechanisms. However, they did not show impaired suppression of alcohol-related memories. Instead, they exhibited increased functional connectivity between brain regions involved in memory suppression when attempting to suppress these memories, likely reflecting heightened attention toward intrusive alcohol-related thoughts and compensatory mechanisms for potential inhibitory control deficits. Similar to alcohol-dependent individuals, binge drinkers also showed impaired suppression of non-alcohol-related memories, which was accompanied by reduced connectivity between inhibitory control and memory networks, suggesting a broader deficit in inhibitory mechanisms. Taken together, these studies indicate that individuals with problematic alcohol use patterns exhibit impairments in memory suppression abilities, particularly in relation to alcohol-related memories. However, further research is needed to better understand the mechanisms underlying these suppression deficits in population with dependent-like behaviors.

## Memory suppression as a novel approach in substance use disorders treatment

The relationship between persistent drug-related memories and the emergence of craving—as described both in the preoccupation/anticipation stage of addiction and in the EI theory of desire—raises a compelling question: could targeting these memories and enhancing the ability to inhibit them offer a novel approach to breaking this cycle? Persistent, maladaptive drug-related memories pose a major challenge to maintaining abstinence, and interventions aimed at addressing these memories have been proposed as promising strategies for addiction treatment (Lee et al., 2005; Noël, 2023). However, to date, no study has investigated the potential impact of strengthening the capacity to suppress drug-associated memories in individuals with SUDs (Almeida-Antunes et al., 2022).

Interestingly, recent evidence indicates that training individuals to suppress negative thoughts improves mental health outcomes in conditions like anxiety and PTSD by reducing repetitive, intrusive thinking (Mamat and Anderson, 2023). Given that recurrent drug-related thoughts seem to be a hallmark in addiction, enhancing memory suppression abilities may similarly reduce the strength and persistence of these maladaptive memories. Additionally, training focused on attentional and executive functions, particularly when tailored to substance-related cues, has been shown to improve cognitive functions and clinical symptoms in SUDs (Bartsch et al., 2016; Nardo et al., 2022; Stein et al., 2023; Verdejo-García, 2016; Verdejo-García et al., 2024; Wiers, 2018), suggesting that drug-specific memory suppression training could significantly impact outcomes, including reducing craving and relapse (Figure 1B).

One commonly used, yet conceptually distinct, method for managing substance-related thoughts is known as *thought suppression* (Wenzlaff and Wegner, 2000). While this technique typically involves instructing individuals to avoid thinking about certain topics—often through vague or general directives—the memory suppression approach differs both in the type of mental content being targeted and in the nature of the suppression strategy. For example, interventions based on thought suppression in addiction contexts include instructions such as: “*Try not to think about smoking. If you do happen to have thoughts about smoking this week, please, try to suppress them*” or “*For the next 5 minutes, please do everything you can to not think about alcohol (…) However, if you should have such a thought, please make a checkmark on this sheet of paper”* (Erskine et al., 2010; Klein, 2007). In contrast, memory suppression—as conceived within the TNT framework—involves a goal-directed, item-specific effort to inhibit memory retrieval in response to specific cues. For instance, when presented with the word cue “*foam*,” participants are instructed to prevent the associated target image—such as people clinking beer glasses—from coming to mind, using a trained direct suppression strategy (Simeonov et al., 2022). Crucially, participants typically engage in repeated attempts to block retrieval, allowing them to improve control over intrusive content over time (Nardo and Anderson, 2024). This progressive improvement is reflected in the decreasing frequency of intrusions across suppression attempts: they occur frequently at first (around 60%), but tend to diminish with practice (~30%), reflecting the so-called intrusion-control effect (Levy and Anderson, 2012). In contrast, thought suppression paradigms usually rely on general avoidance instructions without strategic guidance or practice. These methodological differences are important, as they may account for the divergent outcomes typically associated with each approach. Whereas thought suppression has frequently been linked to ironic rebound effects and increased salience of the suppressed material (Wegner and Erber, 1992; Moss et al., 2015), recent work has questioned the generality of these findings, suggesting that such effects may stem from ambiguities in the instructions and from the interference caused by multitasking or cognitive load during suppression attempts (Mamat et al., 2024). By comparison, memory suppression tasks offer clear, reproducible instructions and engage executive mechanisms to disrupt retrieval processes at the mnemonic level, leading to suppression-induced forgetting (Anderson and Hulbert, 2021) and attenuation of the emotional or motivational salience of the suppressed content (Hu et al., 2017). As such, memory suppression constitutes a more structured and empirically supported form of inhibitory control (Wessel and Anderson, 2024), with promising implications for disrupting the memory-craving-relapse cycle in addiction.

Additionally, while models such as *desire thinking* (Caselli and Spada, 2016) emphasize the role of elaborative and metacognitive processes in sustaining craving, the memory suppression approach for addictive behaviors differs in two fundamental respects: it targets an earlier stage of the craving process, namely the episodic memory reactivations that often precede elaboration, and it involves the active suppression of the memory or mental image itself, rather than the modulation of cognitive elaboration or metacognitive beliefs about thinking.

Although promising, memory suppression training as a treatment for SUDs is still in its early stages. To evaluate its potential clinical impact, further research is needed to assess the type and degree of impairment (if any) in memory suppression mechanisms among individuals with SUDs, and to determine whether enhancing this ability can effectively reduce craving and relapse risk. At present, there is encouraging evidence that interventions targeting maladaptive or unwanted memories could offer an innovative therapeutic pathway (Almeida-Antunes et al., 2024b; Joormann et al., 2009; Mary et al., 2020; Nishiyama and Saito, 2022; Noël, 2023; Mamat and Anderson, 2023), although the generalization of these lab-based interventions and the durability of their effects over time remain to be systematically assessed (Fawcett et al., 2024). These approaches have the potential to complement existing strategies by addressing a crucial yet underexplored dimension of the addiction cycle, opening new avenues for more comprehensive and effective treatments.
